# Rural–urban and educational gradients in head and neck cancer incidence in Finland from 1977 to 2021

**DOI:** 10.2340/1651-226X.2025.43391

**Published:** 2025-08-12

**Authors:** Rayan Nikkilä, Heidi Ryynänen, Aaro Haapaniemi, Nea Malila, Janne Pitkäniemi, Karri Seppä, Antti Mäkitie

**Affiliations:** a Department of Otorhinolaryngology – Head and Neck Surgery, University of Helsinki and HUS Helsinki University Hospital, Helsinki, Finland; b Research Program in Systems Oncology, Faculty of Medicine, University of Helsinki, Helsinki, Finland; c Finnish Cancer Registry, Institute for Statistical and Epidemiological Cancer Research, Helsinki, Finland; d Health Sciences Unit, Faculty of Social Sciences, Tampere University, Tampere, Finland; e Department of Public Health, Faculty of Medicine, University of Helsinki, Helsinki, Finland; f Division of Ear, Nose and Throat Diseases, Department of Clinical Sciences, Intervention and Technology, Karolinska Institutet and Karolinska Hospital, Stockholm, Sweden

**Keywords:** Head and neck neoplasms, urban health, socioeconomic factors, incidence, human papillomavirus infections

## Abstract

**Background and purpose:**

Rural–urban differences in head and neck cancer (HNC) incidence remain understudied, especially in Europe. Changes over time in risk factors, such as smoking and human papillomavirus status, may be reflected in alterations of HNC incidence by subsite, educational level, and urbanity.

**Material and methods:**

Incidence rate ratios (IRR) – adjusted for age, calendar period, educational and urbanization level, and region – and age-standardized HNC incidence per 100,000 person-years were estimated by sex, subsite, levels of education and urbanization over 5-year periods from 1977 to 2021. We estimated the average annual percent change in incidence and IRRs between levels of urbanization and education using Poisson regression.

**Results:**

A lower incidence of oral cavity (IRR 0.82, 95% confidence interval [CI]: 0.73–0.93 for 2007–2021), oropharyngeal (0.75, 0.65–0.87), and nasopharyngeal cancer (0.43, 0.25–0.75) was noted among rural men when compared with urban men. Semi-urban men also showed lower incidences than urban men. Similarly, a lower incidence of oropharyngeal cancer (OPC) was observed among rural (IRR 0.62, 95% CI: 0.47–0.80) and semi-urban women (0.79, 0.63–0.99). Additionally, our study indicates that the rates of OPC and oral cavity cancer are increasing across all educational and urbanization levels. The rise in OPC is particularly notable since 1997–2001, especially among urban populations, in both men and women.

**Interpretation:**

While a higher prevalence of risk factors among urban populations may explain the differences noted across the different urbanization levels, the reasons for the increasing trends across all strata remain unclear.

## Introduction

Most head and neck cancers (HNCs) can be attributed to well-known risk factors, such as tobacco and alcohol consumption [1]. Furthermore, human papillomavirus (HPV) – most commonly transmitted through sexual activities – is implicated in the onset of certain HNCs, particularly oropharyngeal cancer (OPC) [2], with several countries having witnessed a shift in the ratio of HPV-positive/HPV-negative OPCs (HPV^+^ OPC / HPV^-^ OPC) over the last decades [3, 4]. In Europe, HPV infection has been reported to be more frequent in urban populations than in rural areas [5, 6]. Moreover, higher socioeconomic status, usually linked to higher educational attainment, has been associated with an increased incidence of HPV^+^ OPC [7], suggestive of higher rates of HPV^+^ OPC among urban populations, where higher educational attainment is typically more prevalent [8]. However, while higher education is associated with greater HPV-related OPC risk, lower educational attainment remains a risk factor for overall HNC incidence [9].

Numerous studies have reported rural-urban differences in cancer incidence rate [10–15]. However, one major caveat is that many have focused primarily on cancer mortality, which complicates interpretation. While differences in mortality between urban and rural populations may arise from variations in cancer incidence, healthcare access and disease stage at diagnosis may affect survival rates and bias the incidence estimates. Additionally, the prevalence of risk factors often varies across socioeconomic groups. Differences in cancer rates between rural and urban populations may simply reflect socioeconomic differences, which many studies did not account for.

Rural-urban differences in HNC incidence rates remain understudied, especially in Europe, and have not yet been investigated in Finland. Moreover, changes over time in risk factors, such as smoking prevalence, are likely reflected in HNC incidence, including variations by cancer subsite, educational level, and rural-urban regions. Notably, in the context of HPV-related HNCs, a demographic shift in OPC incidence may be observed. This is due to the majority of OPC cases in Finland and other Western countries being increasingly related to HPV, which tends to affect different population groups compared to traditional risk factors like smoking and alcohol use [16]. Our register-based cohort study aims to evaluate subsite-specific differences and trends in nationwide HNC incidence rates between rural and urban areas and across educational levels in Finland.

## Materials and methods

This study combines data from the Finnish Cancer Registry (FCR), the Digital and Population Data Service Agency’s Population Information System, and Statistics Finland. The FCR includes all new primary cancers diagnosed in Finland since 1953 with complete follow-up data until death or emigration. The linkage of these data was done using Finland’s unique personal identity codes.

### Study population

Data on all patients with HNC aged 25 years or over at diagnosis – as higher educational attainment is typically not completed before this age – were obtained from the FCR and linked with the Population Information System, which includes data on individuals’ birth and death dates, sex, and residential address. Only the first primary HNC diagnosed for each individual was included. Patients’ residence was determined based on the home municipality at the start of the calendar year of cancer diagnosis. We assessed HNC incidence in relation to educational attainment level – primary, secondary, and higher – based on individual-level information from Statistics Finland, and in relation to the urbanization level of municipalities – categorized as rural, semi-urban, or urban – based on the proportion of people living in urban settlements and the population of the largest urban settlement (https://stat.fi/en/luokitukset/kuntaryhmitys/). Individuals whose degrees were completed abroad or were unknown are classified under the primary education category. Regions were defined based on the five collaborative areas for healthcare and social welfare of Finland: Southern, Western, Inland, Northern, and Eastern (https://stat.fi/en/luokitukset/yhteistyoalue/yhteistyoalue_1_20240101). No data were missing for any of the included variables.

### Cancer data

HNCs were defined as malignant neoplasms of any histology of the lip (ICD-O-3: C00.0-C00.9), oropharynx (C01.9, C02.4, C05.1, C05.2, C09.0-C10.0, C10.2-C10.9), oral cavity (C02.0-C02.3, C02.8-C05.0, C05.8-C06.9), salivary glands (C07-C08.9), pharynx (C11.0-C14.8), nasal cavity and middle ear (C30.0, C30.1), paranasal sinuses (C31.0-C31.9), and larynx and epiglottis (C10.1, C32.0-C32.9). Of note, cancers of the inner lip (C00.3, C00.4, C00.5) were also classified as lip cancer due to previous coding systems and classification criteria. We limited our study to cancers diagnosed from January 1^st^, 1977 to December 31^st^, 2021.

### Statistical analyses

We calculated age-standardized (Finland 2014) HNC incidence per 100,000 person-years by sex and subsite within the Finnish population aged 25 years and older in 5-year periods from 1977–1982 to 2017–2021. The incidence rates were stratified by educational level and urbanization level. Details of the Finnish population are given in Table 1. Cancer incidence was modeled using Poisson regression to assess trends in incidence over time and to compare incidence across the levels of urbanization and education. The model included the main effects of age (5-year groups from 25–29 to 80–84 years and 85 years and older), calendar year (log-linear), region (Southern, Western, Inland, Northern, and Eastern Finland), urbanization level (urban, semi-urban, rural), and educational level (primary, secondary, higher). We estimated the average annual percentage change (APC) in incidence across educational and urbanization levels and used the Davies’ test to detect if a statistically significant change occurred in the APC. Changes in incidence rates are presented as absolute changes (cases per 100,000) unless otherwise specified, and APCs represent relative changes. Additionally, we estimated adjusted incidence rate ratios (IRRs) for the levels of urbanization and education in periods 1977–2021 and 2007–2021, separately. Heterogeneity in the IRRs was assessed using the likelihood ratio test.

**Table 1:** Characteristics of the study population in Finland by education and urbanization level, stratified by sex, age group, calendar period and region.

Variable	Primary education						Secondary education						Higher education							
	Urban	%	Semi-urban	%	Rural	%	Urban	%	Semi-urban	%	Rural	%	Urban	%	Semi-urban	%	Rural	%	Total	%
**Sex**																				
Men	407,426	(24)	127,603	(8)	149,324	(9)	404,006	(24)	107,599	(6)	99,916	(6)	313,510	(18)	51,387	(3)	36,844	(2)	1,697,618	(100)
Women	499,929	(27)	139,305	(8)	151,286	(8)	393,785	(21)	98,519	(5)	91,274	(5)	369,429	(20)	64,432	(3)	47,340	(3)	1,855,299	(100)
**Age group**																				
25–44	261,203	(18)	61,431	(4)	62,847	(4)	436,385	(30)	104,954	(7)	92,553	(6)	336,882	(23)	54,582	(4)	38,324	(3)	1,449,162	(100)
45–64	330,239	(26)	100,149	(8)	113,441	(9)	265,293	(21)	75,263	(6)	72,360	(6)	253,457	(20)	46,080	(4)	34,329	(3)	1,290,612	(100)
65–84	279,874	(38)	93,335	(13)	110,027	(15)	89,707	(12)	24,372	(3)	24,630	(3)	86,164	(12)	14,235	(2)	10,823	(1)	733,165	(100)
85+	36,040	(45)	11,993	(15)	14,297	(18)	6405	(8)	1528	(2)	1647	(2)	6436	(8)	921	(1)	709	(1)	79,977	(100)
**Calendar period**																				
1977–1991	1,157,547	(36)	354,388	(11)	411,163	(13)	533,733	(17)	130,988	(4)	123,837	(4)	360,421	(11)	61,117	(2)	45,783	(1)	3,178,978	(100)
1992–2006	888,967	(25)	264,013	(7)	296,272	(8)	828,402	(23)	215,748	(6)	204,224	(6)	666,388	(19)	117,673	(3)	86,917	(2)	3,568,605	(100)
2007–2021	675,552	(17)	182,322	(5)	194,397	(5)	1,031,236	(26)	271,619	(7)	245,510	(6)	1,022,009	(26)	168,665	(4)	119,854	(3)	3,911,165	(100)
**Region**																				
Southern	432,729	(33)	42,369	(3)	35,897	(3)	367,995	(28)	30,209	(2)	22,509	(2)	356,678	(27)	20,446	(2)	11,385	(1)	1,320,218	(100)
Western	144,340	(24)	59,074	(10)	62,891	(10)	115,439	(19)	46,617	(8)	38,957	(6)	89,546	(15)	29,998	(5)	19,957	(3)	606,819	(100)
Inland	126,938	(22)	66,060	(12)	50,339	(9)	117,476	(21)	47,366	(8)	32,274	(6)	91,937	(16)	24,558	(4)	15,017	(3)	571,966	(100)
Eastern	113,790	(19)	53,553	(9)	91,657	(16)	106,132	(18)	43,983	(7)	56,562	(10)	76,948	(13)	23,074	(4)	22,284	(4)	587,984	(100)
Northern	89,559	(19)	45,852	(10)	59,826	(13)	90,748	(19)	37,943	(8)	40,888	(9)	67,829	(15)	17,742	(4)	15,542	(3)	465,929	(100)
Total	907,355	(26)	266,908	(8)	300,611	(8)	797,791	(22)	206,118	(6)	191,190	(5)	682,939	(19)	115,819	(3)	84,184	(2)	3,552,915	(100)

### Analytical assumptions

**Figure 1:**
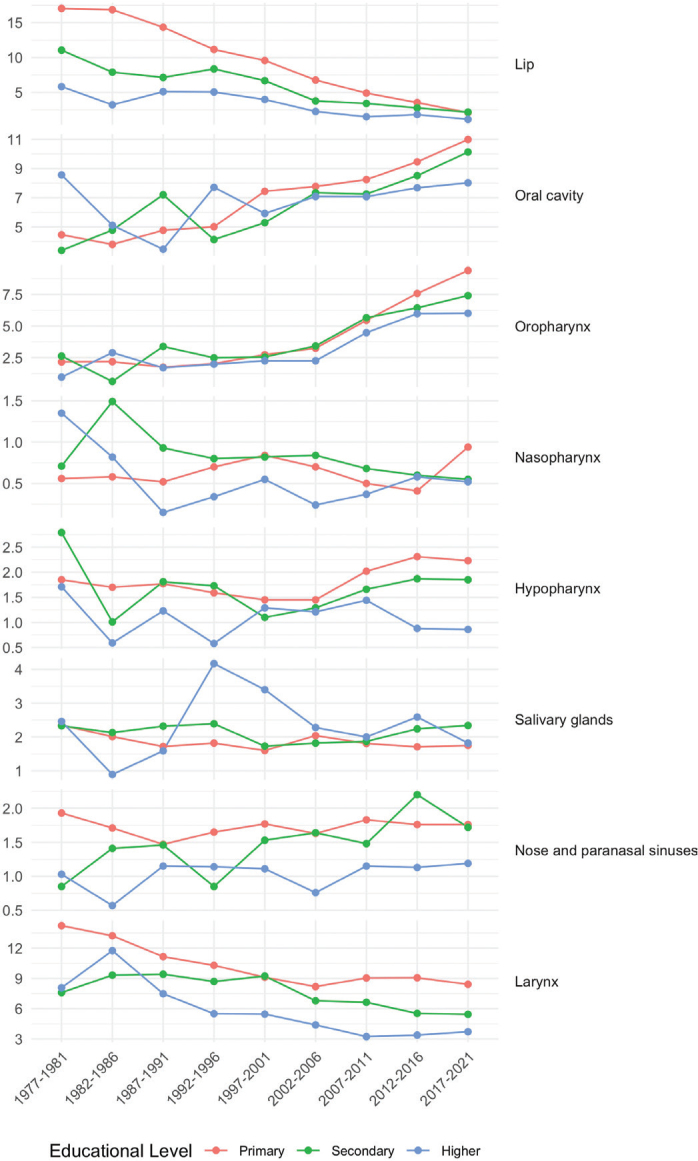
Age-standardized head and neck cancer incidence per 100,000 person-years among men in 5-year periods from 1977–1981 to 2017–2021 by cancer subsite and educational level.

**Figure 2:**
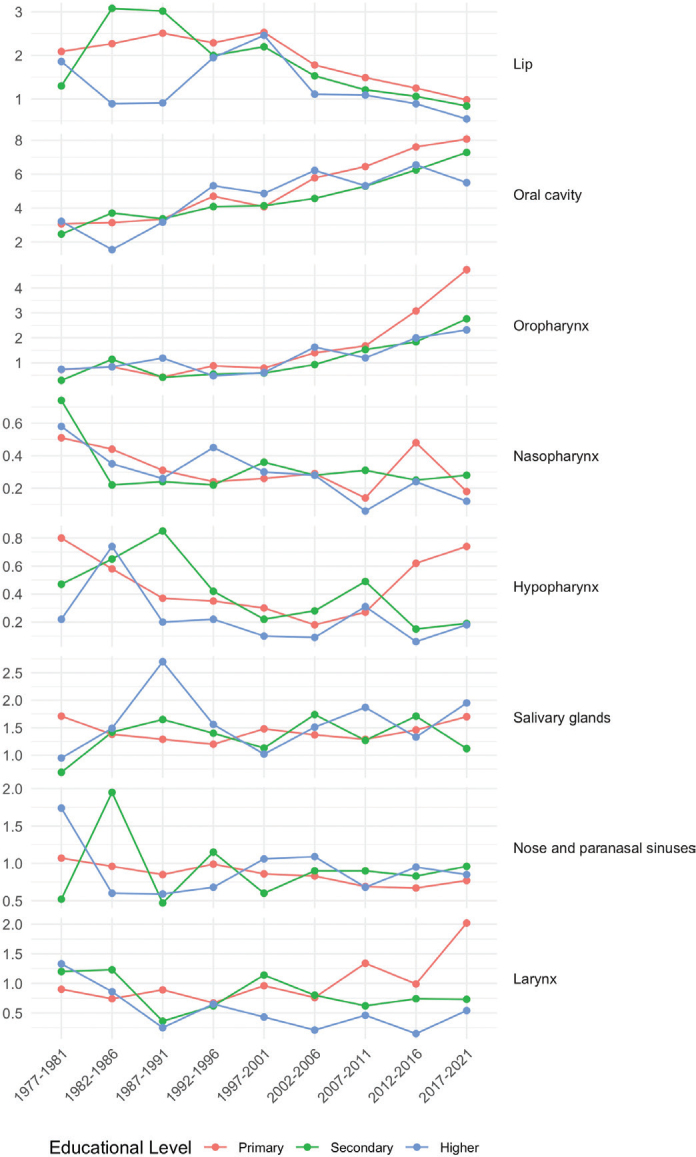
Age-standardized head and neck cancer incidence per 100,000 person-years among women in 5-year periods from 1977–1981 to 2017–2021 by cancer subsite and educational level.

**Figure 3:**
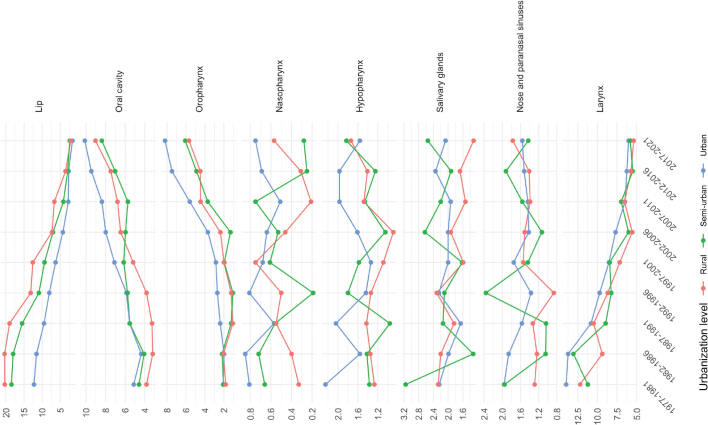
Age-standardized head and neck cancer incidence per 100,000 person-years among men in 5-year periods from 1977–1981 to 2017–2021 by cancer subsite and urbanization level.

**Figure 4:**
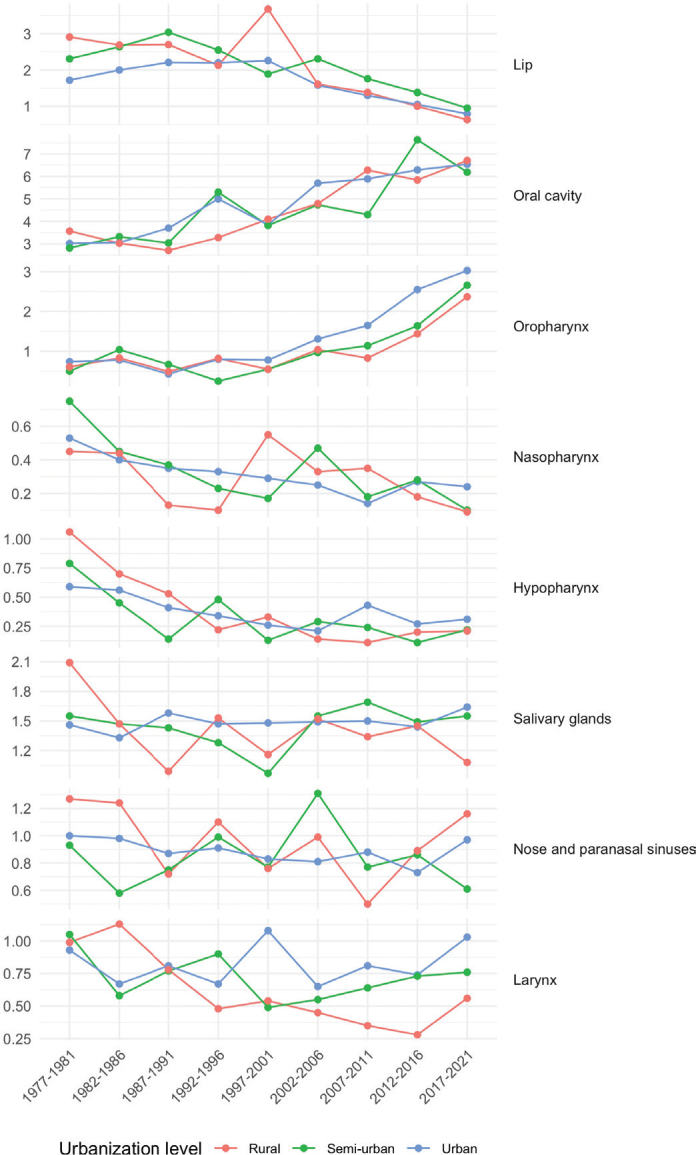
Age-standardized head and neck cancer incidence per 100,000 person-years among women in 5-year periods from 1977–1981 to 2017–2021 by cancer subsite and urbanization level.

We assumed that educational and urbanization levels may, independently and jointly, influence the incidence of HNC. In our models, we adjusted for age, calendar year, education, urbanization, and region to estimate the independent association of each exposure with HNC incidence. When the educational level was the exposure, we adjusted for urbanization and region to account for area-level contextual factors – such as environmental exposures, access to healthcare, and regional variation in risk behaviors – that might confound the education–HNC incidence association. Thus, we estimate the associations of education with HNC incidence among people living in similar types of areas (urban, semi-urban, rural). Conversely, when urbanization was the exposure, we adjusted for educational level and region to separate the influence of socioeconomic status from urbanization – that is, what is the difference in incidence between urban and rural areas, if education were held constant? We acknowledge, however, that the relationships among education, urbanization, and region are complex and potentially bidirectional. For example, early-life urbanicity may shape educational opportunities, but educational attainment may also influence residential choices. In such cases, adjusting for potential mediators (education when estimating urbanization effects or urbanization when estimating educational effects) may underestimate the total effect. We included regions as an adjustment variable to account for potential geographical variation in healthcare access, diagnostic practices, environmental exposures, and risk factor prevalence that are not fully captured by the urbanization classification alone. Our adjusted models therefore estimate the independent associations of educational level and urbanization, while our incidence rates (Figures 1–4) adjusted for age only, reflect total differences across strata.

Analyses were done separately by sex and subsite (excl. middle ear due to the small number of cases) and were conducted using the statistical program R version 4.3.2, and popEpi package version 0.4.11. Research permission for the study was granted by Statistics Finland (Permits TK/2743/07.03.00/2023 and TK-53-675-17).

## Results

In Finland, from 1977 to 2021, 28,794 new HNC cases were diagnosed: 19,355 (67%) in men and 9,439 (33%) in women (Supplement table).

In the analysis of the entire study period (1977–2021) – after adjusting for age, calendar year, region, and urbanization level – differences in incidence were observed among men and women between educational levels for all HNC subsites, except cancers of the nasopharynx, salivary glands, and nose and paranasal sinuses (Table 2). The IRRs for these cancers remained stable throughout the study period, with men and women with higher education consistently showing the lowest incidences (IRR < 1).

**Table 2:** Age-standardized incidence rates (ASIR) per 100,000 person-years and incidence rate ratios (IRR) with 95% confidence intervals (95% CI) for head and neck cancer subsites in Finland (for the latest 15-year period 2007–2021 and the whole study period 1977–2021) by educational level (reference level: primary education), adjusted for age, calendar period, urbanization level, and region.

	2007–2021				1977–2021			
	ASIR	IRR and 95% CI		*p*	ASIR	IRR and 95% CI		*p*
**Men**								
**Lip**								
Primary education	3.56				9.62			
Secondary education	2.66	0.79	(0.65, 0.95)		3.97	0.66	(0.59, 0.73)	
Higher education	1.44	0.48	(0.38, 0.61)	**<0.001**	2.36	0.41	(0.36, 0.47)	**<0.001**
**Oral cavity**								
Primary education	9.44				6.56			
Secondary education	8.93	0.96	(0.87, 1.06)		7.82	0.96	(0.89, 1.04)	
Higher education	7.66	0.74	(0.67, 0.83)	**<0.001**	7.08	0.78	(0.72, 0.85)	**<0.001**
**Oropharynx**								
Primary education	7.28				3.58			
Secondary education	6.63	0.85	(0.76, 0.96)		5.22	0.89	(0.81, 0.98)	
Higher education	5.57	0.69	(0.61, 0.78)	**<0.001**	4.07	0.69	(0.62, 0.77)	**<0.001**
**Nasopharynx**								
Primary education	0.60				0.63			
Secondary education	0.60	1.07	(0.73, 1.58)		0.70	1.13	(0.88, 1.44)	
Higher education	0.49	0.77	(0.50, 1.20)	0.270	0.50	0.81	(0.61, 1.07)	0.067
**Hypopharynx**								
Primary education	2.20				1.73			
Secondary education	1.84	0.88	(0.71, 1.08)		1.71	0.89	(0.76, 1.05)	
Higher education	1.04	0.44	(0.33, 0.57)	**<0.001**	1.07	0.50	(0.41, 0.61)	**<0.001**
**Salivary glands**								
Primary education	1.76				1.87			
Secondary education	2.17	1.15	(0.93, 1.43)		2.11	1.12	(0.97, 1.31)	
Higher education	2.10	1.13	(0.90, 1.41)	0.401	2.28	1.22	(1.05, 1.42)	**0.035**
**Nose and paranasal sinuses**								
Primary education	1.80				1.69			
Secondary education	1.78	0.93	(0.73, 1.17)		1.63	0.89	(0.75, 1.05)	
Higher education	1.15	0.60	(0.46, 0.80)	**0.001**	1.09	0.60	(0.49, 0.73)	**<0.001**
**Larynx**								
Primary education	8.93				10.41			
Secondary education	5.76	0.68	(0.61, 0.76)		6.74	0.74	(0.68, 0.79)	
Higher education	3.51	0.36	(0.31, 0.42)	**<0.001**	4.58	0.43	(0.39, 0.47)	**<0.001**
**Women**								
**Lip**								
Primary education	1.25				1.96			
Secondary education	1.03	0.90	(0.70, 1.16)		1.42	0.88	(0.76, 1.04)	
Higher education	0.79	0.65	(0.46, 0.90)	**0.026**	1.05	0.71	(0.58, 0.88)	**0.005**
**Oral cavity**								
Primary education	7.24				4.66			
Secondary education	6.37	0.94	(0.85, 1.05)		5.36	0.93	(0.86, 1.02)	
Higher education	5.78	0.78	(0.69, 0.89)	**<0.001**	5.33	0.84	(0.76, 0.92)	**0.001**
**Oropharynx**								
Primary education	2.88				1.18			
Secondary education	2.10	0.69	(0.57, 0.84)		1.49	0.73	(0.62, 0.86)	
Higher education	1.92	0.57	(0.47, 0.71)	**0.001**	1.58	0.66	(0.56, 0.79)	**<0.001**
**Nasopharynx**								
Primary education	0.26				0.30			
Secondary education	0.28	1.22	(0.66, 2.26)		0.29	1.10	(0.78, 1.55)	
Higher education	0.15	0.70	(0.34, 1.43)	0.197	0.24	0.97	(0.66, 1.44)	0.770
**Hypopharynx**								
Primary education	0.48				0.43			
Secondary education	0.26	0.59	(0.36, 0.99)		0.31	0.85	(0.61, 1.18)	
**Salivary glands**								
Primary education	1.43				1.40			
Secondary education	1.36	0.92	(0.73, 1.17)		1.37	0.94	(0.81, 1.11)	
Higher education	1.71	1.15	(0.90, 1.47)	0.169	1.63	1.13	(0.95, 1.33)	0.133
**Nose and paranasal sinuses**								
Primary education	0.70				0.86			
Secondary education	0.89	1.10	(0.82, 1.50)		0.86	1.01	(0.82, 1.23)	
Higher education	0.84	1.07	(0.77, 1.49)	0.812	0.88	1.04	(0.83, 1.31)	0.959
**Larynx**								
Primary education	1.37				0.92			
Secondary education	0.70	0.52	(0.38, 0.70)		0.76	0.75	(0.61, 0.93)	
Higher education	0.40	0.24	(0.16, 0.36)	**<0.001**	0.43	0.37	(0.27, 0.50)	**<0.001**

*P*-value assesses heterogeneity in IRRs across education levels.

Bold values indicate that p-value is less than 0.05 and heterogeneity in IRRS was considered statistically significant.

Among men, all educational levels experienced a decrease in the incidence of lip and laryngeal cancers over time (Figure 1), with the most pronounced decreases among those with primary education: from 1977–1981 to 2017–2021, the incidence of lip cancer dropped gradually from 17.05 to 2.09 per 10^5^, and laryngeal cancer from 14.22 to 8.41 per 10^5^. The incidence of lip cancer among men with primary education leveled off with that of other educational levels.

The incidence of oral cavity cancer and OPC has been progressively rising similarly among men across all educational levels since 1997–2001. From 1977–1981 to 2017–2021, the incidence of oral cavity cancer increased from 7.44 to 10.99 per 10^5^ among men with primary education, from 5.29 to 10.13 per 10^5^ among men with secondary education, and from 5.92 to 8.02 per 10^5^ among men with higher education.

The incidence of OPC started to increase after 1997–2001 from 2.74 to 9.39 per 10^5^ by 2017–2021 among men with primary education (APC 7.0% from 1997 to 2021, 95% confidence interval [CI]: 5.9–8.0), from 2.56 to 7.41 per 10^5^ among men with secondary education (APC 4.6% from 1977 to 2021, 3.9–5.4), and from 2.26 to 6.01 per 10^5^ among men with higher education (APC 4.7% from 1977 to 2021, 3.8–5.7) (Table 3).

**Table 3:** Annual percentage change in head and neck cancer incidence during 1977–2021 among men and women by cancer subsite and educational level, adjusted for age, region, and educational level.

Subsite	Men								
	Annual percentage change 1977–2021 and 95%-confidence interval								
	Primary education			Secondary education			Higher educational		
Lip	−1.7%	1977–1989	(−3.1, −0.4)	−5.1%	1977–2021	(−5.8, −4.4)	−4.6%	1977–2021	(−5.5, −3.6)
	−5.7%	1990–2021	(−6.2, −5.1)						
Oral cavity	2.7%		(2.4, 3.1)	2.4%		(1.8, 2.9)	1.7%		(1.0, 2.3)
Oropharynx	0.2%	1977–1996	(−1.8, 2.3)	4.6%	1977−2021	(3.9, 5.4)	4.7%	1977−2021	(3.8, 5.7)
	7.0%	1997–2021	(5.9, 8.0)						
Nasopharynx	0.1%	1977–2021	(−1.0, 1.2)	−1.3%	1977–2021	(−2.7, 0.2)	−6.0%	1977–2003	(−9.9, −2.0)
								2004–2021	(−2.4, 10.3)
Hypopharynx	−0.9%	1977–2001	(−2.4, 0.7)	1.2%	1977–2021	(0.0, 2.4)	−0.1%	1977–2021	(−1.5, 1.5)
	2.6%	2002–2021	(0.5, 4.8)						
Salivary glands	−0.4%		(−1.0, 0.3)	0.4%		(−0.6, 1.3)	−0.2%		(−1.1, 0.8)
Nose and sinuses	−0.1%		(−0.8, 0.5)	1.0%		(−0.2, 2.2)	0.0%		(−1.5, 1.4)
Larynx	−2.4%	1977–2003	(−3.0, −1.9)	−1.5%	1977–2021	(−2.1, −1.0)	−3.3%	1977–2012	(−4.3, −2.3)
	0.2%	2004–2021	(−0.9, 1.2)				3.0%	2013–2021	(−2.3, 8.5)
Subsite	Women								
	Annual percentage change 1977–2021 and 95%−confidence interval								
	Primary education			Secondary education			Higher educational		
Lip	0.8%	1977–1998	(−0.6, 2.2)	10.0%	1977–1988	(−4.6, 26.8)	2.4%	1977–1999	(−2.9, 8.1)
	−4.5%	1999–2021	(−5.8, −3.2)	−4.0%	1989–2021	(−5.5, −2.6)	−6.9%	2000–2021	(−10.0, −3.6)
Oral cavity	2.2%		(1.9, 2.6)	2.6%		(2.0, 3.2)	1.4%		(0.7, 2.2)
Oropharynx	0.5%	1977–1999	(−1.9, 2.9)	6.0%	1977–2021	(4.6, 7.4)	4.6%	1977–2021	(3.1, 6.1)
	8.4%	2000–2021	(6.5, 10.3)						
Nasopharynx	−2.7%		(−4.1, −1.2)	−1.0%		(−3.1, 1.1)	−2.4%		(−4.7, 0.1)
Hypopharynx	−5.5%	1977–2003	(−7.7, −3.3)	−2.1%	1977–2021	(−4.2, −0.1)	−2.2%	1977–2021	(−5.2, 1.0)
	6.3%	2004–2021	(0.8, 12.2)						
Salivary glands	−0.1%		(−0.7, 0.6)	0.4%	1977–2021	(−0.6, 1.5)	0.3%		(−0.8, 1.4)
Nose and sinuses	−1.0%		(−1.7, −0.2)	0.9%	1977–2021	(−0.5, 2.3)	0.1%		(−1.4, 1.7)
Larynx	0.1%	1977–2011	(−1.1, 1.2)	−0.3%	1977–2021	(−1.7, 1.1)	−7.7%	1977–2003	(−12.5, −2.6)
	8.4%	2012–2021	(1.0, 16.5)				5.2%	2004–2021	(−1.9, 12.8)

Regarding hypopharyngeal cancer, men with primary and secondary education showed a gradual increase in incidence after 1997–2001.

As in men, while the incidence of lip cancer has gradually decreased, the incidence of oral cavity and OPCs has progressively risen among women across all educational groups (Figure 2). From 1977–1981 to 2017–2021, oral cavity cancer incidence increased from 3.07 to 8.07 per 10^5^ among women with primary education, from 2.47 to 7.28 per 10^5^ among those with secondary education, and from 3.22 to 5.50 per 10^5^ among those with higher education.

The incidence of OPC increased after 1997–2021, especially among women with primary education, with an APC of 7.0% (95% CI: 5.9–8.0) between 1997 and 2021. Among women with primary education, an increasing trend in the incidence of hypopharyngeal (APC of 6.3% from 2004 to 2021, 95% CI: 0.8–12.2) and laryngeal cancers was also seen (APC of 8.4% from 2012 to 2021, 1.0–16.5).

In the analysis of the entire study period (1977–2021) – after adjusting for age, calendar period, region, and educational level – differences in incidences among men between urbanization levels were observed for all cancer subsites, except for salivary gland, sinonasal (tai nose and paranasal sinus) cancers (Table 4). Men in semi-urban and rural areas, compared to urban men, had lower incidences of oral cavity, oropharyngeal, nasopharyngeal, and laryngeal cancers (IRR < 1) throughout the study period. Women in semi-urban and rural areas had lower incidences of OPC (IRR < 1) compared to urban women.

**Table 4:** Age-standardized incidence rates (ASIR) per 100,000 person-years and incidence rate ratios (IRR) with 95% confidence intervals (95% CI) for head and neck cancer subsites in Finland (for the latest 15-year period 2007–2021 and the whole study period 1977–2021) by urbanization level (reference level: urban) adjusted for age, calendar period, educational level, and region.

	2007–2021				1977–2021			
	ASIR	IRR and 95% CI		*p*	ASIR	IRR and 95% CI		*p*
**Men**								
**Lip**								
Urban	2.31				5.27			
Semi-urban	3.13	1.21	(0.99–1.48)		8.27	1.30	(1.19, 1.41)	
Rural	4.00	1.41	(1.17, 1.71)	**0.002**	10.42	1.42	(1.32, 1.54)	**<0.001**
**Oral cavity**								
Urban	9.42				7.66			
Semi-urban	7.21	0.79	(0.70, 0.89)		6.13	0.84	(0.77, 0.92)	
Rural	7.78	0.82	(0.73, 0.93)	**<0.001**	5.74	0.80	(0.73, 0.87)	**<0.001**
**Oropharynx**								
Urban	7.16				4.74			
Semi-urban	5.00	0.78	(0.67, 0.89)		2.99	0.72	(0.64, 0.81)	
Rural	4.90	0.75	(0.65, 0.87)	**<0.001**	2.91	0.72	(0.64, 0.81)	**<0.001**
**Nasopharynx**								
Urban	0.65				0.70			
Semi-urban	0.42	0.58	(0.36, 0.93)		0.50	0.70	(0.53, 0.92)	
Rural	0.37	0.43	(0.25, 0.75)	**0.001**	0.45	0.61	(0.45, 0.82)	**0.001**
**Hypopharynx**								
Urban	1.84				1.73			
Semi-urban	1.52	0.91	(0.70, 1.18)		1.45	0.89	(0.75, 1.07)	
Rural	1.55	0.93	(0.72, 1.22)	0.733	1.31	0.80	(0.66, 0.96)	**0.040**
**Salivary glands**								
Urban	2.10				2.04			
Semi-urban	2.20	1.02	(0.81, 1.28)		2.18	1.13	(0.97, 1.32)	
Rural	1.50	0.69	(0.53, 0.90)	**0.011**	1.79	0.91	(0.78, 1.07)	0.072
**Nose and paranasal sinuses**								
Urban	1.50				1.56			
Semi-urban	1.66	1.10	(0.84, 1.43)		1.55	0.97	(0.81, 1.16)	
Rural	1.48	0.96	(0.73, 1.28)	0.708	1.37	0.85	(0.71, 1.03)	0.229
**Larynx**								
Urban	6.28				8.47			
Semi-urban	6.19	0.97	(0.85, 1.11)		7.91	0.91	(0.84, 0.98)	
Rural	5.91	0.92	(0.80, 1.05)	0.453	7.63	0.82	(0.76, 0.88)	**<0.001**
**Women**								
**Lip**								
Urban	1.03				1.56			
Semi-urban	1.36	1.22	(0.94, 1.58)		2.03	1.19	(1.04, 1.37)	
Rural	1.00	0.87	(0.65, 1.16)	0.112	2.01	1.07	(0.93, 1.23)	**0.046**
**Oral cavity**								
Urban	6.23				5.03			
Semi-urban	6.10	1.00	(0.88, 1.13)		4.81	1.00	(0.91, 1.09)	
Rural	6.37	0.99	(0.87, 1.12)	0.987	4.57	0.94	(0.86, 1.03)	0.426
**Oropharynx**								
Urban	2.44				1.49			
Semi-urban	1.83	0.79	(0.63, 0.99)		1.10	0.80	(0.67, 0.96)	
Rural	1.51	0.62	(0.47, 0.80)	**<0.001**	0.96	0.73	(0.60, 0.89)	**0.001**
**Nasopharynx**								
Urban	0.22				0.29			
Semi-urban	0.19	0.95	(0.47, 1.93)		0.30	1.07	(0.75, 1.52)	
Rural	0.20	1.04	(0.51, 2.14)	0.979	0.29	0.97	(0.68, 1.40)	0.908
**Hypopharynx**								
Urban	0.33				0.36			
Semi-urban	0.20	0.62	(0.32, 1.19)		0.31	0.84	(0.59, 1.19)	
Rural	0.17	0.45	(0.20, 1.01)	0.057	0.37	1.00	(0.72, 1.39)	0.590
Urban	1.53				1.49			
Semi-urban	1.58	1.03	(0.80, 1.31)		1.45	0.98	(0.84, 1.15)	
Rural	1.28	0.83	(0.63, 1.09)	0.347	1.38	0.92	(0.78, 1.08)	0.602
**Nose and paranasal sinuses**								
Urban	0.86				0.86			
Semi-urban	0.76	0.96	(0.68, 1.36)		0.84	0.97	(0.79, 1.20)	
Rural	0.85	1.06	(0.75, 1.50)	0.898	0.94	1.09	(0.89, 1.33)	0.646
**Larynx**								
Urban	0.87				0.84			
Semi-urban	0.71	0.84	(0.59, 1.21)		0.71	0.88	(0.70, 1.10)	
Rural	0.38	0.41	(0.25, 0.67)	**<0.001**	0.61	0.71	(0.55, 0.91)	**0.017**

*P*-value assesses heterogeneity in IRRs across urbanization levels.

Bold values indicate that p-value is less than 0.05 and heterogeneity in IRRS was considered statistically significant.

Lip cancer showed a gradual decrease in incidence across all levels of urbanization (Figures 3 and 4). Initially, the highest incidence was observed among rural men and women; however, the difference has gradually diminished over time.

Conversely, oral cavity and OPC exhibited an increasing trend in incidence across all urbanization levels among men and women. Notably, the incidence of OPC increased after 1992–1996, rising from 2.69 to 8.21 per 10^5^ (2017–2021) among urban men, from 1.05 to 6.08 per 10^5^ among semi-urban men, and from 1.23 to 5.68 per 10^5^ among rural men. 1987–1991. Since 1987–1991, urban men have consistently exhibited the highest incidence rates, with the difference widening further after 2007–2011. OPC exhibited the highest APCs: 6.0% (95% CI: 4.6–7.4) from 1989 to 2021 among rural men, 7.3% (5.6–9.1) from 1995 to 2021 among semi-urban men, and 5.5% (4.7–6.2) from 1997 to 2021 among urban men (Table 5). Similarly, among women, between 1997–2001 and 2017–2021, the incidence of OPC increased from 0.78 to 3.03 per 10^5^ among urban women (APC of 6.5% from 1990 to 2021, 95% CI: 5.6–7.5), from 0.55 to 2.66 per 10^5^ among semi-urban women (APC of 9.2% from 1990 to 2021, 6.0–12.6), and from 0.55 to 2.37 per 10^5^ among rural women (APC of 3.8% from 1977 to 2021, 2.3–5.3).

**Table 5:** Annual percentage change in head and neck cancer incidence during 1977–2021 among men and women by cancer subsite and urbanization level, adjusted for age, region, and educational level.

Subsite	Men								
	Annual percentage change 1977–2021 and 95%-confidence interval								
	Urban			Semi-urban			Rural		
Lip	−2.8%	1977–1992	(−4.2, −1.4)	−1.4%	1977–1986	(−5.2, 2.5)	1.7%	1977–1986	(−2.2, 5.8)
	−5.5%	1993–2021	(−6.2, −4.7)	−5.5%	1987–2021	(−6.3, −4.7)	−5.8%	1987–2021	(−6.4, −5.1)
Oral cavity	2.5%		(2.1, 2.8)	2.0%		(1.4, 2.6)	2.8%		(2.2, 3.5)
Oropharynx	2.3%	1977–1996	(0.2, 4.5)	−2.2%	1977–1994	(−7.0, 2.9)	−3.9%	1977–1988	(−12.3, 5.4)
	5.5%	1997–2021	(4.7, 6.2)	7.3%	1995–2021	(5.6, 9.1)	6.0%	1989–2021	(4.6, 7.4)
Nasopharynx	−0.5%		(−1.5, 0.4)	−1.5%		(−3.4, 0.4)	−0.5%		(−2.5, 1.6)
Hypopharynx	0.5%		(−0.1, 1.2)	0.7%		(−0.5, 2.0)	1.3%		(0.0, 2.6)
Salivary glands	0.1%		(−0.4, 0.7)	−0.1%		(−1.1, 0.9)	−1.3%		(−2.3, −0.2)
Nose and sinuses	−0.1%		(−0.8, 0.6)	0.4%		(−0.8, 1.6)	0.7%		(−0.5, 1.9)
Larynx	−1.7%		(−2.0, −1.5)	−1.6%		(−2.1, −1.1)	−1.5%		(−2.0, −1.0)
Subsite	Women								
	Annual percentage change 1977–2021 and 95%−confidence interval								
	Urban			Semi-urban			Rural		
Lip	2.0%	1977–1997	(0.1, 3.9)	−1.9%	1977–2021	(−2.8, −1.1)	0.2%	1977–1999	(−2.1, 2.6)
	−4.5%	1998–2021	(−5.8, −3.3)				−6.7%	2000–2021	(−9.3, −4.0)
Oral cavity	2.8%	1977–2006	(2.1, 3.6)	2.2%	1977–2021	(1.5, 2.8)	−3.3%	1977–1987	(−10.0, 3.8)
	1.0%	2007–2021	(−0.1, 2.2)				3.1%	1988–2021	(2.2, 4.0)
Oropharynx	−3.4%	1977–1989	(−9.6, 3.3)	−1.9%	1977–1998	(−7.2, 3.7)	3.8%	1977–2021	(2.3, 5.3)
	6.5%	1990–2021	(5.6, 7.5)	9.2%	1999–2021	(6.0, 12.6)			
Nasopharynx	−2.1%		(−3.4, −0.8)	−3.1%		(−5.4, −0.7)	−1.4%		(−3.8, 1.0)
Hypopharynx	−1.2%		(−2.4, 0.0)	−2.6%		(−5.0, −0.2)	−5.4%		(−7.7, −3.0)
Salivary glands	0.2%		(−0.4, 0.8)	0.5%		(−0.6, 1.6)	−0.8%		(−1.9, 0.4)
Nose and sinuses	−0.3%		(−1.1, 0.5)	−0.2%		(−1.6, 1.3)	−1.1%		(−2.4, 0.3)
Larynx	1.1%	1977–2021	(0.3, 1.9)	0.3%	1977–2021	(−1.2, 1.9)	−3.7%	1977–2015	(−5.8, −1.6)
							25.6%	2016–2021	(−15.8, 87.2)

While the incidence of laryngeal cancer declined over time across all urbanization levels among men, an increasing trend was observed among urban women (APC of 1.1% from 1977 to 2021, 0.3–1.9).

## Discussion

Since 1977–1981, in Finland, the incidence of oral cavity and OPCs has progressively risen among men and women of all educational and urbanization levels. The increase in OPC is particularly evident after 1997–2001, with urban men and women consistently showing the highest incidence, even after adjusting for educational level, age, calendar period and region. Additionally, men with primary and secondary education had an increasing trend in hypopharyngeal cancer incidence. Among women with primary education, hypopharyngeal and laryngeal cancer incidences have increased from 2002 to 2006. To the best of our knowledge, this is the first study assessing rural–urban and educational differences in HNC incidence in the Nordic countries.

In Finland, smoking prevalence among men gradually decreased from 51% in 1972 to 20% in 2022, while among women, it first rose from 11% in 1972 to 23% by 2002 before decreasing to 14% in 2022 [17, 18]. Among men, smoking rates declined across all educational levels but remained consistently higher among those with lower educational attainment compared to those with higher education. Among women, smoking rates increased among those with lower education attainment until the early 2000s, followed by a gradual decline, while smoking among highly educated women peaked in the late 1980s and then gradually decreased [19]. Similarly, alcohol consumption in the Finnish population tripled between 1960 and 2005, then gradually decreased. Historically, women have consumed less alcohol than men, reflected in the lower incidence rates of hypopharyngeal cancer among women. However, in the last three decades, there has been a gender convergence in drinking behaviors. Heavy use of alcohol has been more common among men and women with lower education levels than among those with higher education [20].

Higher rates of smoking and alcohol use are likely contributors to the elevated incidence of oral cavity, hypopharyngeal, and laryngeal cancers among those with primary education. Declining smoking parallels falling laryngeal cancer rates in men across all educational and urbanization levels, while rising rates among women with primary education since 2002–2006 may reflect the rise in smoking among this group until the early 2000s and its long latency effect [19, 21]. The higher incidence of laryngeal cancer among rural populations over the study period (1977–2021) may also reflect historical smoking disparities, which narrowed over time, as indicated by the disappearance of urban–rural differences in laryngeal cancer incidence after 2007 [22]. Since 2002–2006, hypopharyngeal cancer incidence has risen among men and women with primary education, likely due to higher exposure to tobacco and alcohol [20, 23].

Higher total and heavy (more than once a month) alcohol consumption in urban areas may partially explain the associated higher incidence of oral cavity cancer [20, 23]. However, the incidence has been increasing across all education and urbanization levels despite the overall decreasing trends in smoking and alcohol consumption, mirroring results from other studies [24]. As oral cavity cancer is not strongly associated with HPV, other factors – such as diet – may play a key role in the increasing incidence [25]. A meta-analysis reported that a diet rich in fruits and vegetables is associated with a reduced risk of oral cavity cancer, whereas high consumption of processed meat is associated with an increased risk [26, 27]. The rise in ultra-processed food consumption in high-income countries may thus contribute to the increased risk of oral cavity cancer observed. Moreover, urbanization no longer is a strong risk factor for unhealthy diets at the population level, as dietary habits have become more uniform [22, 28]. This might explain the observed increasing trends in oral cavity cancer incidence irrespective of education and urbanization.

The incidence of OPC is increasing among both older and younger men in several countries [29]. The proportion of HPV^+^ OPC has increased over time, with European cases rising from 40% before 2000 to 59% by 2000–2004 [30]. In Finland, a significant increase in the frequency of p16-positive – a marker for HPV infection – head and squamous cell carcinoma tumors was observed in a hospital-based cohort of 135 patients, rising from 22% in 1990–1999 to 41% during 2000–2007, with 85% of the p16-positive tumors being oropharyngeal squamous cell carcinomas [31]. In our study, the increase in OPC incidence was most pronounced in urban men and women. This contrasts with U.S. data, where HPV-related cancer incidence has grown mainly in rural areas [10]. While air pollution, specifically PM2.5, has been linked to oral and pharyngeal cancer risk in the U.S. [32], pollutant levels have declined in Helsinki since 1994, making it an unlikely driver in Finland [33]. Recently administered prophylactic HPV vaccines for early adolescent boys and girls are expected to progressively decrease the incidence of HPV^+^ OPC [34].

The decline in lip cancer incidence in Finland mirrors similar trends observed in other countries and is likely due to the increased awareness of ultraviolet radiation, the use of sunscreen and protective clothing, and enhanced equipment (such as tractor cabins) in the work field [24]. Additionally, the decrease in smoking prevalence has also most likely contributed to the declining incidence trend, as smoking is a known risk factor for lip cancer [17, 35]. Higher incidence among rural men may reflect greater sun exposure from outdoor occupations, while women’s use of sunscreen and lip cosmetics may contribute to their lower rates [36]. While initially rural and semi-urban men had higher lip cancer incidence rates compared with urban men, these rates have since converged.

Beyond individual-level risk factors, several contextual changes may also affect incidence trends. First, advances in diagnostic techniques over the recent decades may have elevated cancer detection rates. Previously non-symptomatic indolent tumors may nowadays be overdiagnosed, as has been reported for example for breast cancer [37]. Secondly, misclassification and changes in cancer classification and coding systems can also influence incidence rates, particularly for cancer subsites with overlapping anatomical definitions, such as the oral cavity and oropharynx or oral cavity and lip. While the whole lip was previously classified as one site, according to the newest TNM classification TNM of 2017, inner lip malignant tumors – located on the labial mucosa – are nowadays classified as oral cavity cancer. This reclassification may have resulted in a transient surge in the incidence of oral cavity cancer but still fails to explain the ongoing increasing trend.

Educational attainment levels have also increased in Finland during the study period: in 1977–1991 15% of ≥ 25-year-olds had completed higher education, rising to 34% in 2007–2021. Over time, as more people complete higher education, the overall patterns of risk factor exposure and healthcare use shift across birth cohorts. These changes would result in different patterns of HNC risk and detection, influencing incidence rates and the distribution of HNCs across the population. Finally, the role of sex- and age-specific interactions in risk factor exposure could potentially explain some of the observed differences in incidence across urbanization levels, especially for OPC. As previously noted, the increasing incidence has been strongly linked to HPV infection, which is influenced by sexual behavior patterns that differ by both sex and age. Men are more likely to acquire persistent oral HPV infections and younger birth cohorts may also have different sexual behavior patterns – such as earlier sexual debut and a higher number of oral sex partners – contributing to higher HPV exposure [38]. These sex- and age-related differences in risk factors could result in diverging incidence trends over time and may partly explain why the incidence of OPC is particularly pronounced among urban populations.

The major strength of the current study is the FCR’s high coverage and diagnostic accuracy, capturing 96% of solid tumors [39]. However, a notable limitation is the lack of data on etiological factors, such as smoking, alcohol use, and HPV status. Finally, as the study is based only on Finnish data, its geographical validity may be limited when applied to other countries with different healthcare systems, risk factor distributions, and demographics.

To conclude, a higher incidence of oral cavity, oropharyngeal, hypopharyngeal, and laryngeal cancers is noted among men and women with primary education. While the incidence of oral cavity cancer and OPC is highest among urban men when compared to semi-urban and rural men, our study also indicates that these rates are increasing across all educational and urbanization levels. This rise in OPC has been particularly notable since 1997–2001. While the increased incidence of oral cavity and OPC among urban populations is likely explained by the higher prevalence of known risk factors and variations in healthcare use, the causes for the increasing trends remain unclear. Understanding national trends in the incidence of HNC by urbanization levels can help in planning public health interventions for cancer prevention. These interventions could include efforts to reduce modifiable risk factors such as smoking and unprotected sexual activity and promote HPV vaccination. Clinicians can also use this information to better understand the risks faced by their patient populations.

## Supplementary Material



## Data Availability

Raw data sharing is not available due to privacy restrictions.
