# Supplementary material for: Rural–urban and educational gradients in head and neck cancer incidence in Finland from 1977 to 2021
Source: Acta Oncol. 2025 Aug 12;64:43391. doi: 10.2340/1651-226X.2025.43391 (PMC13064435; doi:10.2340/1651-226X.2025.43391)
Supplement: Supplementary file 1 [file AO-64-43391-s1.pdf]

Supplementary material has been published as submitted. It has not been copyedited, or typeset by Acta Oncologica

Suppl. Table 1. Number of new head and neck cancers diagnosed in Finland from 1977 to 2021 by subsite, educational, and urbanization level.

Men

Period		Lip	Oral cavity	Oropharynx	Nasopharynx	Hypopharynx	Salivary glands	Nose and paranasal sinuses	Larynx
**2007-2021**	**Educational level**								
	Primary	423	899	643	51	214	188	175	878
	Secondary	162	882	672	64	175	189	149	519
	Higher	86	562	443	37	75	152	80	233
	**Urbanization**								
	Rural	183	362	234	16	75	69	70	283
	Semi-urban	142	343	250	21	73	103	79	294
	Urban	346	1,638	1,274	115	316	357	255	1,053
	**All**	671	2,343	1,758	152	464	529	404	1,630
**1977-2021**	**Educational level**								
	Primary	3,144	2,127	1,177	208	573	601	547	3,441
	Secondary	443	1,325	884	130	258	325	240	994
	Higher	249	901	581	78	131	294	139	527
	**Urbanization**								
	Rural	1,191	692	361	57	156	208	166	918
	Semi-urban	865	691	360	62	160	242	172	886
	Urban	1,780	2,970	1,921	297	646	770	588	3,158
	**All**	3,836	4,353	2,642	416	962	1,220	926	4,962

Women

Period		Lip	Oral cavity	Oropharynx	Nasopharynx	Hypopharynx	Salivary glands	Nose and paranasal sinuses	Larynx
**2007-2021**	**Educational level**								
	Primary	244	942	254	20	45	190	109	135
	Secondary	91	633	228	29	28	139	89	75
	Higher	48	447	186	16	14	152	74	32
	**Urbanization**								
	Rural	61	329	68	10	8	65	44	19
	Semi-urban	85	341	94	10	11	86	43	39
	Urban	237	1,352	506	45	68	330	185	184
	**All**	383	2,022	668	65	87	481	272	242
**1977-2021**	**Educational level**								
	Primary	1,098	2,310	503	135	194	656	431	410
	Secondary	206	955	295	61	58	271	154	142
	Higher	112	683	246	44	26	256	122	56
	**Urbanization**								
	Rural	306	656	130	41	53	194	138	84
	Semi-urban	293	676	147	43	41	202	117	97
	Urban	817	2,616	767	156	184	787	452	427
	**All**	1,416	3,948	1,044	240	278	1,183	707	608
